# Calcium Regulation and Bone Mineral Metabolism in Elderly Patients with Chronic Kidney Disease

**DOI:** 10.3390/nu5061913

**Published:** 2013-05-29

**Authors:** Vickram Tejwani, Qi Qian

**Affiliations:** Division of Nephrology, Department of Medicine, Mayo Clinic College of Medicine, Rochester, MN 55905, USA; E-Mail: tejwani.vickram@mayo.edu

**Keywords:** calcium homeostasis, aging, chronic kidney disease, mineral and bone disorder, vascular calcification, secondary hyperparathyroidism

## Abstract

The elderly chronic kidney disease (CKD) population is growing. Both aging and CKD can disrupt calcium (Ca^2+^) homeostasis and cause alterations of multiple Ca^2+^-regulatory mechanisms, including parathyroid hormone, vitamin D, fibroblast growth factor-23/Klotho, calcium-sensing receptor and Ca^2+^-phosphate product. These alterations can be deleterious to bone mineral metabolism and soft tissue health, leading to metabolic bone disease and vascular calcification and aging, termed CKD-mineral and bone disorder (MBD). CKD-MBD is associated with morbid clinical outcomes, including fracture, cardiovascular events and all-cause mortality. In this paper, we comprehensively review Ca^2+^ regulation and bone mineral metabolism, with a special emphasis on elderly CKD patients. We also present the current treatment-guidelines and management options for CKD-MBD.

## 1. Introduction

Defects in calcium (Ca^2+^) homeostasis and bone mineral metabolism are major causes of morbidity and mortality in elderly chronic kidney disease (CKD) patients, a steadily growing population worldwide [1]. In this paper, the role of Ca^2+^ and its dysregulation in elderly CKD is given special consideration, incorporating the most recent advances in pathophysiology. We describe (1) the physiology of Ca^2+^ homeostasis, (2) CKD-associated Ca^2+^ and bone mineral dysregulation and (3) treatment-guidelines and therapeutic options for elderly CKD patients. It serves as a useful reference for healthcare providers caring for CKD patients. Clear understanding of the pathophysiology and appropriate management of elderly CKD patients can reduce morbidity and mortality.

## 2. Calcium Homeostasis and Defects in Aging and CKD

The average daily diet of a normal adult contains roughly 1000 mg of Ca^2+^. Approximately 300 mg of ingested Ca^2+^ is absorbed from the intestine, promoted by vitamin D [2]. In the circulation, Ca^2+^ exists in three forms: ionized (~51%, Ca^2+^), protein-bound (~40%, primarily albumin-bound) and complexed (~10%); the ionized portion is functional [2]. The protein-bound portion can be influenced by blood pH: increased by alkalemia and reduced by acidemia. Bone mineral metabolism influences Ca^2+^ concentration by releasing or absorbing circulating Ca^2+^. When in balance, bone Ca^2+^ absorption equals bone Ca^2+^ resorption; hence, absorbed dietary Ca^2+^ is excreted by both the colon (~100–150 mg/day) and kidneys (~150–200 mg/day). 

**Figure 1:**
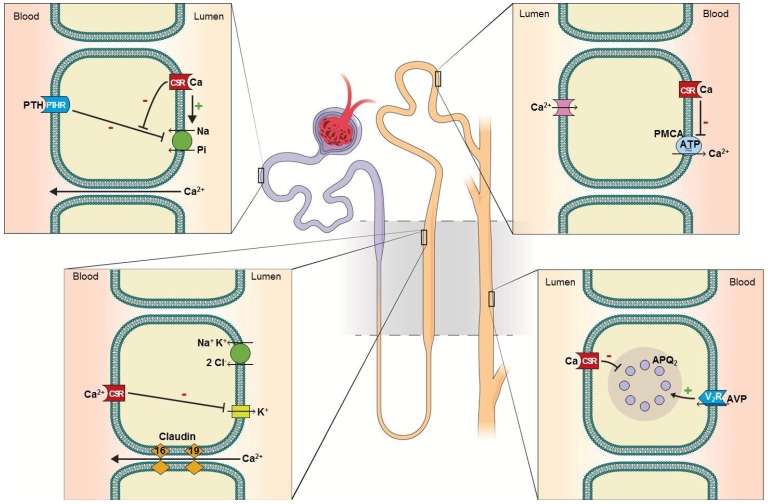
Calcium reabsorption and calcium-sensing receptor in the nephron. (**A**) Proximal tubule: 50%–60% of filtered Ca^2+^ is reabsorbed paracellularly. Luminal calcium-sensing receptor (CSR) activation counteracts parathyroid hormone (PTH)-mediated P_i_ excretion, thereby promoting P_i_ conservation [11]. (**B**) Thick ascending limb: 30%–35% of filtered Ca^2+^ is reabsorbed paracellularly. Basolateral CSR inhibits potassium excretion via renal outer medullary potassium channel (ROMK), diminishing Ca^2+^ (as well as magnesium) reabsorption. Diminished potassium exit also reduces NaCl reabsorption via NKCl_2_, analogous to the effect of loop diuretics [12]. (**C**) Distal convoluted tubule: 10% of filtered Ca^2+^ is reabsorbed transcellularly. Basolateral CSR inhibits plasma membrane calcium ATPase (PMCA), thereby inhibiting transcellular Ca^2+^ reabsorption [10]. (**D**) Collecting duct: in the principle cells, activation of CSR inhibits retention of aquaporin-2 in the luminal-surface of the plasma membrane, thereby inhibiting antidiuretic hormone-mediated water conservation, causing renal water wasting [13,14]. In the intercalated cells, CSR promotes the activity of proton pump (H^+^-ATPase), enhancing urine acidification, thereby minimizing the risk of Ca^2+^ × P_i_ supersaturation [15].

In the kidney, Ca^2+^ is freely filtered through the glomeruli. Fifty to 60% of filtered Ca^2+^ is reabsorbed in the proximal tubule via the paracellular pathway coupled to sodium reabsorption (Figure 1A). The reabsorption is enhanced by volume contraction and reduced by volume expansion [3]. Thirty to 35% of filtered Ca^2+^ is reabsorbed in the thick ascending limb of the loop of Henle via the paracellular pathway (Figure 1B) [4]. In this segment, serum Ca^2+^, loop diuretics and the claudin family of proteins influence Ca^2+^ reabsorption. Hypercalcemia activates the basolateral calcium-sensing receptor (CSR) inhibiting potassium excretion via the renal outer medullary potassium channel (ROMK), diminishing the positive luminal potential, leading to reduction in Ca^2+^ (and magnesium) reabsorption (reviewed in [5]). Loop diuretics inhibit sodium-potassium-chloride (NKCl_2_) cotransporter, reducing ROMK-mediated potassium exit, thereby diminishing Ca2+ (and magnesium) reabsorption. Claudin-16 and claudin-19 reside in the paracellular space and facilitate Ca^2+^ reabsorption. Mutations in these proteins impair Ca^2+^ reabsorption and cause the autosomal recessive familial hypomagnesemia with hypercalciuria and nephrocalcinosis [6]. The remaining 10% of the filtered Ca^2+^ is reabsorbed at the distal renal tubules through a transcellular mechanism (Figure 1C) [3]. TRPV5, a subfamily V member of the transient receptor potential (TRP) cation channels, acts as the luminal-surface Ca^2+^ receptor [7]. TRPV5-mediated Ca^2+^ influx is promoted by Klotho (an anti-aging molecule, Box 1) [8,9] and Ca^2+^ exits via basolateral plasma membrane calcium ATPase (PMCA). CSR activationinhibits PMCA activity, thereby inhibiting transcellular Ca^2+^ reabsorption [10]. Through a yet to be fully elucidated mechanism, Ca^2+^ reabsorption in this tubular segment is enhanced by parathyroid hormone (PTH), thiazide diuretics and alkalemia [3].

Aging is well-known to be associated with a progressive decline in glomerular filtration rate (GFR). By age 75, 30% of the glomeruli are obsolete, due to sclerosis [16], and the glomerular clearance (of inulin) drops from 122.8 to 65.3 mL/min/1.73 m^2^ between the ages of 20–29 and ≥80 [17]. Moreover, tubular excretory capacity, independent of GFR, is reduced by nearly a half (54.6 and 30.8 mg/min). Thus, age-related decline in kidney function can substantially diminish kidney reserve. In parallel with aging, the prevalence of CKD has also been steadily rising (CKD definition: Table 1). In the United States, CKD prevalence in those ≥20 years of age has increased from 14.5% in 1988–1994 to 16.8% in 1999–2004, affecting approximately one in six adults [18]. A vast majority of CKD is caused by chronic diabetes and hypertension.

**Box 1. Klotho.**

- Loss-of-function mutation in Klotho causes premature aging phenotype [19], which led to the initial discovery of the gene and appropriate naming of the gene: Klotho, the Goddess of life.
- Klotho encodes a single-pass transmembrane protein, also termed Klotho, that is expressed primarily in kidney and parathyroid gland [20].
- Klotho couples with FGF23 receptor to form a high-affinity receptor complex for FGF23 and mediates FGF23-induced phosphaturia [21].
- Klotho promotes Ca^2+^ conservation through stimulating TRPV-5 in the distal convoluted tubule [22].
- Extracellular domain of Klotho can be enzymatically cleaved and shed into the extracellular space, becoming a secreted form of Klotho.
- The secreted form of Klotho exerts pleiotropic activities, including suppression of growth factor signaling [23], oxidative stress [24], inflammation [25] and fibrosis [26].
- Klotho is regarded as an anti-aging molecule [23].

**Table 1:** The Kidney Disease Outcomes Quality Initiative (KDOQI)—(2002) definition of CKD. GFR, glomerular filtration rate.

* Stage 1: Kidney damage with GFR greater than 90 mL/min/1.73 m^2^. * Stage 2: Mild reduction in GFR, 60–89 mL/min/1.73 m^2^. Stage 3: Moderate reduction in GFR, 30–59 mL/min/1.73 m^2^. Stage 4: Severe reduction in GFR, 15–29 mL/min/1.73 m^2^. Stage 5: Kidney failure, GFR less than 15 mL/min/1.73 m^2^ or dialysis.

* Note: Stage 1 and 2 CKD are not diagnosed based on GFR. There should be other markers of kidney damage, including abnormalities in urine studies, including active urine sediment or proteinuria or abnormalities on kidney imaging studies.

Both aging and CKD can cause alterations in Ca^2+^ homeostasis. Age-related decline in kidney function can reduce renal expression of Klotho, leading to diminished vitamin D activation through fibroblast growth factor-23 (FGF23) signaling and impairment of renal tubular Ca^2+^ reabsorption [22]. CKD, in addition to aging, can cause a further steep drop in Klotho [27], elevation in FGF23 [28] and more impairment of renal Ca^2+^ conservation [22,29]. A strong correlation of CKD severity with the magnitude of alterations in FGF23, active vitamin D, PTH, Klotho and bone mineral metabolism has been demonstrated in a number of studies [27,28,30]. Moreover, CKD patients exhibit less regulated intestinal Ca^2+^ absorption. Spiegel *et al*. compared differences in Ca^2+^ balance between healthy individuals and late CKD patients (estimated GFR [eGFR]: 20–33 mL/min/1.73 m^2^) on daily intakes of 800 mg *vs*. 2000 mg elemental Ca^2+^ for nine days [31]. Compared to normal individuals, CKD patients demonstrated greater non-regulated intestinal Ca^2+^ absorption. Low Ca^2+^ intake (800 mg) in CKD patients resulted in a negative net Ca^2+^ balance, while high Ca^2+^ intake (2000 mg) resulted in a positive net Ca^2+^ balance. These findings indicate that CKD confers a high-risk of Ca^2+^ imbalance associated with dietary Ca^2+^ intake.

Age-related alterations in Ca^2+^ homeostasis, with or without CKD, can lead to serious adverse outcomes. Foley *et al*., showed significant associations between circulating Ca^2+^, phosphate (P_i_) and calcium-phosphate (Ca^2+^ × P_i_) product with age, female sex and low eGFR (among others) in 15,732 U.S. community-dwelling individuals (mean age: 54.2 years; mean eGFR: 93.1 mL/min/1.73 m^2^) followed prospectively over 12.6 years. Notably, after correcting for age and eGFR differences, elevated Ca^2+^ concentration was associated with higher risk of stroke and elevated Ca^2+^ × P_i_ product associated with higher risk of both stroke and death [32]. A meta-analysis of randomized, placebo-controlled trials found Ca^2+^ supplementation, with or without vitamin D, to be positively associated with cardiovascular events [33]. More recently, a prospective population-based study involving 388,229 individuals in the U.S. for a mean follow-up of 12 years also showed a clear association between Ca^2+^ supplementation and cardiovascular disease and mortality; the association, however, was significant only in men, not in women [34]. In men, there was a U-shaped relation between Ca^2+^ intake and cardiovascular risk. It was postulated that in women, the results might have been obscured, because of a much younger age in initiation of Ca^2+^ supplementation. The abrupt increase in serum Ca^2+^ in men, due to late Ca^2+^ initiation, can potentially contribute to the adverse outcomes [35]. Consistently, another recent prospective longitudinal study from Europe involving 61,433 women (Swedish mammography cohort) with a median follow-up of 19 years showed significant increase in cardiovascular and all-cause mortality in patients with a high dietary and supplemental Ca^2+^ intake (≥1400 mg/day) [36]. Interestingly, a non-significant U-shaped trend between Ca^2+^ intake and cardiovascular risk was also observed. Overall, it seems apparent that high Ca^2+^ intake can adversely impact cardiovascular disease and mortality. 

Chronic hypocalcemia or net negative Ca^2+^ balance, conversely, is known to be associated with osteopenia and osteoporosis, resulting in fractures, morbidity and mortality [37,38]. Studies have shown approximately 18% of hospitalized patients [39] and 85% of intensive care unit patients [40] are hypocalcemic. Kolb *et al*. studied 94 post-menopausal females with a mean age of 74.9 years who presented with distal radial fractures requiring surgical repair [41]. At presentation, 83.5% were vitamin D-deficient (mean ± SEM: 19.6 ± 21.9 ng/mL; optimal levels >30 ng/mL) and 21.3% hypocalcemic (8.92 ± 1.4 mg/dL; normal range: 8.9–10.1 mg/dL). Ca^2+^ and vitamin D supplementation post-surgery for six weeks improved serum vitamin D, Ca^2+^ and fracture site callus formation. The supplementation also normalized PTH levels in those with PTH elevation. While osteopenia and osteoporosis are much more common in elderly women, primarily related to estrogen reduction, elderly males are not spared. In a prospective study of 178 men, age was associated with a decrease in intestinal Ca^2+^ absorption, related to the co-existing vitamin D reduction [42]. Notably, the magnitude of hypocalcemia in these studies was relatively small, as observed in prior studies [43,44]; however, Ca^2+^ concentration is maintained at the cost of hyper- or hypo-activations in multiple regulatory elements (serum P_i_, vitamin D, PTH, FGF23/Klotho and CSR-mediated signaling) and active bone destruction. All of the elements are heavily influenced, directly or indirectly, by CKD. Below, we review the Ca^2+^ regulatory mechanisms and bone mineral metabolism with special emphasis on pathophysiology relating to aging and CKD.

## 3. Phosphate

Western diet contains 0.8–1.5 g of P_i_ daily. Seventy to 90% of dietary P_i_ is absorbed, a process enhanced by active vitamin D. P_i_ is predominantly distributed in bone and cells with <1% in the circulation. When in balance, net P_i_ mobilization from bone is negligible; therefore, absorbed dietary P_i_ is excreted predominantly by the kidneys. Following glomerular filtration, a large proportion of P_i_ is reabsorbed in the proximal tubules. The reabsorption is regulated by dietary P_i_, PTH, FGF23 and chronic metabolic acidosis, all promoting P_i_ excretion [3]. 

Age-related decline in GFR and tubular excretory capacity reduces renal reserve, predisposing to P_i_ retention. Consistently, elderly patients are found to be at a higher risk for acute P_i_ nephropathy following ingestion of P_i_-containing purgatives [45]. CKD further reduces P_i_ excretion capacity, causing P_i_ retention. FGF23 elevation in early CKD may mitigate P_i_ retention by inhibiting tubular P_i_ reabsorption [46]. In late CKD (eGFR < 40 mL/min/1.73 m^2^), such compensation becomes insufficient and hyperphosphatemia ensues [43,44,47]. Hyperphosphatemia inhibits 1α-hydroxylation of vitamin D and stimulates FGF23 and PTH production and parathyroid hyperplasia. Hyperphosphatemia also reduces Ca^2+^ in the circulation; the resulting hypocalcemia further stimulates PTH synthesis and secretion. All of these factors contribute to the development of secondary hyperparathyroidism and MBD. 

Beyond bone mineral regulation, P_i_ is an emerging key regulator of aging and vascular calcification. Hyperphosphatemia stimulates osteogenic transformation and apoptosis of vascular smooth muscle cells, causing expression of genes promoting matrix mineralization and Ca^2+^ deposition [48,49,50]. Hyperphosphatemia clinically is linked to accelerated vascular calcification and mortality [51,52,53]. Dietary P_i_ restriction in a mouse model ameliorates the premature-aging phenotype [54]. 

## 4. Vitamin D

The major source of vitamin D, without supplementation, is from skin. Cholecalciferol (vitamin D_3_) is produced through conversion of 7-dehydrocholesterol stimulated by ultraviolet radiation in sunlight. A casual 10–20 min of noontime-sun exposure generates an adequate daily requirement of vitamin D [55]. Insufficient sun exposure can cause vitamin D deficiency. Day-to-day western food is a poor source of vitamin D. Consequently, dairy products, such as milk, have been fortified with cholecalciferol or ergocalciferol (vitamin D_2_). Both vitamins D_3_ and D_2_ are converted to 25-hydroxyvitamin D (25[OH]D) via hepatic P-450 enzymes [56]. In the circulation, 25(OH)D binds to vitamin D-binding protein and is further 1α-hydroxylated in the kidney to the highly active 1,25-dihydroxyvitamin D (1,25[OH]_2_D, calcitriol); ~100-fold efficacy of that in 25(OH)D. 25(OH)D and 1,25(OH)_2_D can be converted to inactive 24,25(OH)_2_D in both the liver and kidneys [57]. The conversion to active vitamin D (1,25[OH]_2_D) is highly regulated, promoted by PTH and inhibited by FGF23 and hyperphosphatemia. 

At the cellular level, active vitamin D binds to vitamin D receptor (VDR), dimerizes with the retinoid x-receptor and activates downstream transcriptions [58]. Through this mechanism, vitamin D can be involved in a variety of genomic regulations, including the regulation of hematopoietic cells, muscle function, immunomodulatory function, inflammation and fibrosis, beyond vitamin D’s classic roles in mineral metabolism, *i.e*., promoting intestinal Ca^2+^ and P_i_ absorption and facilitating PTH-mediated bone resorption [59]. The pleiotropic effects of vitamin D have been linked to multiple health benefits, e.g., lowering blood pressure via inhibition of the renin-angiotensin-aldosterone system [60,61,62,63] and lowering the risk of diabetes [64,65], colorectal and breast cancer [66] and infection in immunodeficient patients [67]. Vitamin D deficiency is also found to be associated with poor cognitive function and risk for Alzheimer disease [68], possibly relating to vitamin D’s role in phagocytosing soluble amyloid-β [69]. The survival advantage in individuals with an adequate vitamin D store has been demonstrated in several epidemiological studies [37,70,71,72].

With growing recognition of salutatory effects of vitamin D, the threshold for optimal vitamin D status has been raised to a serum 25(OH)D level of >30 ng/mL (75 nmol/L) [73,74,75]. 25(OH)D levels are chosen as a surrogate for vitamin D status, because of its stability and ease of assaying [76]. Per this definition, over half of the U.S. and European community-dwelling populations are classified as vitamin D-deficient and 40%–50% have levels <20 ng/mL (50 nmol/L) [77,78]. Elderly CKD patients are at a particular risk for vitamin D deficiency. Age reduces skin production [79] and intestinal absorption of vitamin D [80]. Age-related GFR decline reduces 1α-hydroxylation and attendant vitamin D activation. CKD-associated P_i_ retention and FGF23 elevation inhibit 1α-hydroxylase activity, further impairing vitamin D activation [23]. Additionally, proteinuria, often associated with CKD, can increase urine loss of protein-bound 25(OH)D [81]. 

Similar to the observations in the general population, vitamin D deficiency in CKD is associated with increased mortality [82]. In the elderly (≥65 years old), intake of vitamin D (≥800 IU) with Ca^2+^reduces fracture risk by ~14%–30% [83]. Moreover, vitamin D or analogue (paricalcitol) supplementation reduces albuminuria, systemic blood pressure [84] and vascular calcification [85] and improves patient survival with [86] or without [87,88,89] Ca^2+^ cosupplementation. The potential mechanisms of vitamin D-associated survival advantage are an area of active research. A recent study shows that vitamin D or paricalcitol administration, equivalent to the dose used in CKD patients, in mouse (50% and 75%) renal ablation models is associated with an elevated serum and urinary Klotho and reduction in the magnitude of arterial calcification compared with untreated controls [90]. Given the known anti-aging effect of Klotho, it is tempting to speculate that the benefit of vitamin D in CKD might be related to the enhanced Klotho signal. Although there is much more to be learned about the beneficial effects of vitamin D supplementation, the study begins to draw a connection between vitamin D, vascular calcification and aging. It provides support for current practice guidelines, advocating vitamin D repletion for CKD patients.

## 5. Parathyroid Hormone

**Figure 2:**
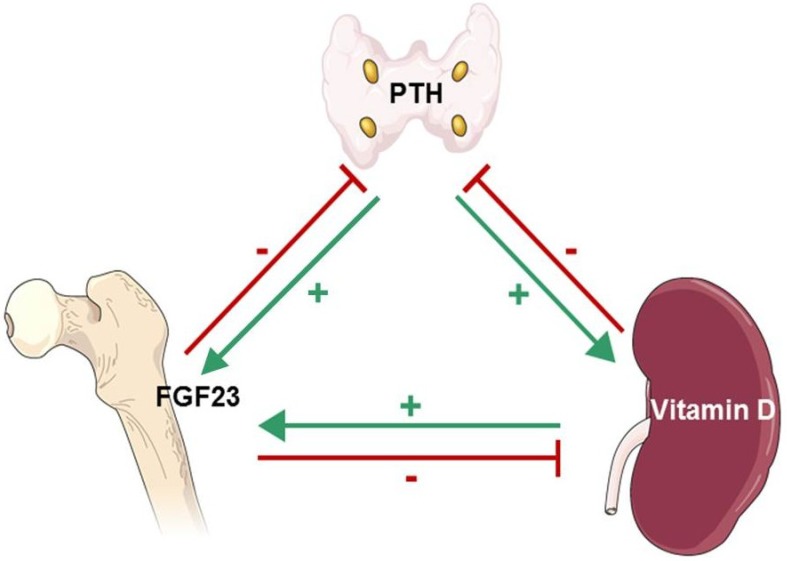
Interplay of PTH, FGF23 and active vitamin D: PTH increases bone FGF23 gene expression [96] (Green, Parathyroid Gland→Bone) and kidney proximal tubule 1α-hydroxylation of 25(OH)D [97] (Green, Parathyroid Gland→Kidney). 1,25(OH)D2 (active vitamin D) binds to parathyroid vitamin D receptor (VDR), inhibiting PTH gene transcription [95] (Red, Kidney→Parathyroid Gland) and stimulates osteoblast and osteoclast FGF23 production [98] (Green, Kidney→Bone). FGF23 increases parathyroid CSR and VDR expression, inhibiting PTH production [99] (Red, Bone→Parathyroid Gland). FGF23 also decreases kidney proximal tubule 1α-hydroxylation, reducing vitamin D activation, and increases kidney (and liver) 24-hydroxylation of 25(OH)D, enhancing vitamin D inactivation [100] (Red, Bone→Kidney).

Intact PTH is an 84-amino acid peptide produced by the parathyroid glands. It has a half-life of ~10 min, and the n-terminal portion is biologically active. PTH is metabolized in the liver, and the resulting inactive c-terminal portion is excreted by the kidneys [91,92]. PTH production is stimulated by hypocalcemia, sensed through CSR [93,94], and inhibited by vitamin D, sensed through VDR [95]. The effector organs of PTH are bone and the kidneys. In bone, PTH receptor is expressed in both osteoblasts and osteoclasts, where, in concert with active vitamin D, it accelerates bone turnover. PTH also increases FGF23 gene expression (Figure 2) [96]. In the kidneys, PTH stimulates 1α-hydroxylation of vitamin D, reduces proximal tubular P_i_ reabsorption and enhances distal tubular Ca^2+^ reabsorption [97].

The elderly population has a higher occurrence of primary hyperparathyroidism, due to benign parathyroid adenoma [101]. CKD exerts an important impact on PTH, as the impairment of P_i_ excretion and hypocalcemia in CKD stimulates PTH synthesis and secretion, causing secondary hyperparathyroidism. Due to the impairment of P_i_ excretion and a degree of bone resistance to PTH in CKD, the reference range of serum PTH for CKD patients is raised to 150–300 pg/mL from <65 pg/mL in the general population, as a higher level of PTH is necessary to maintain adequate bone turnover in CKD. A PTH level >300 pg/mL is an indication for active treatment [102]. Levels <150 pg/mL are considered a sign of adynamic bone disease, and therapies that may suppress PTH (e.g., calcium, vitamin D and its analogues) should be avoided [103,104]. Although secondary hyperparathyroidism is evident in the majority of CKD patients and is associated with the genesis of MBD and MBD-related complications, the direct relation between PTH and cardiovascular complications or cardiovascular disease-related mortality in CKD is unclear and needs to be further studied. 

## 6. Fibroblast Growth Factor-23

FGF23, a member of the phosphatonin family of proteins, is a 251-amino acid peptide [105]. It is produced by osteoclasts and osteoblasts and plays a major role in P_i_ and, indirectly, Ca^2+^ homeostasis [100,106,107]. The production of FGF23 is stimulated by active vitamin D and likely by positive net P_i_ balance [98]. FGF23 binds to its receptor complex (Klotho-FGFR-1) and induces phosphaturia by suppressing luminal-surface expression of sodium**-**P_i_ co-transporters 2A and 2C in the renal proximal tubules [100]. It also suppresses the 1α-hydroxylation of vitamin D by inhibiting 1α-hydroxylase (CYP27B1) activity and by increasing 24-hydroxylase activity, which increases the production of inactive 24,25(OH)_2_D (Figure 2). Thus, FGF23 reduces P_i_ and Ca^2+^. Additionally, FGF23 increases gene expression and protein production of CSR and VDR, reducing parathyroid cell proliferation, attenuating parathyroid function [99]. *In vitro*, high-Ca^2+^ concentrations have been shown to increase both parathyroid Klotho and FGF receptor expression, suggesting that hypercalcemia may promote the effect of FGF23 on the parathyroid glands [108]. FGF23 is inactivated by an endopeptidase. Mutations in the endopeptidase cause hypophosphatemic rickets associated with renal P_i_ wasting and osteomalacia [109]. 

Mesenchymal tumors are seen in elderly and associated with FGF23 over-production and urine P_i_ wasting, hypophosphatemia, 1,25(OH)_2_D deficiency and osteomalacia [110,111]. Rare forms of FGF23 gain-of-function [112] and loss-of-function [113] mutations can cause familial forms of hyper- or hypophosphatemia, vitamin D alterations and soft-tissue injury. A much more common cause of FGF23 elevation, however, is CKD. Even in patients with a mild degree of CKD, FGF23 can be elevated. Westerberg *et al*. reported the observation of transient FGF23 elevation in healthy individuals after kidney donation. The elevation seems to be associated with transient reduction in eGFR and active vitamin D with net positive P_i_ balance. Once the remaining kidney improves its clearance by compensation, FGF23 level returns to normal, along with normalization of P_i_ balance and active vitamin D [99]. Even in early stage CKD, FGF23 elevation is persistent and accompanied by a decline in Klotho, rendering resistance to FGF23-mediated effects. FGF23 elevation in CKD is associated with several adverse outcomes, including: (1) greater risk of end stage renal disease (ESRD) (if eGFR > 30 mL/min/1.73 m^2^) [30]; (2) faster progression to refractory secondary hyperparathyroidism [108]; (3) left ventricular hypertrophy [114,115] and (4) higher mortality rate in dialysis patients [116,117]. Studies using rat CKD (nephritic and 5/6 nephrectomy) models showed that monoclonal-FGF23 antibody injection was associated with a reversal of secondary hyperparathyroidism, increase in serum vitamin D and Ca^2+^ and normalization of bone markers [118,119]. However, kidney function was unchanged, and there was an FGF23-antibody dose-dependent increase in serum P_i_ and aortic calcification [119]. In aggregate, FGF23 inhibition in this model ameliorated MBD, but had no effect on kidney function, vascular calcification and mortality. It would be informative to further investigate whether dietary P_i_ restriction, in addition to FGF23 ablation, alters the final outcome. 

## 7. Calcium Sensing Receptor

CSR, a G-protein coupled receptor activated by Ca^2+^, is abundantly expressed in the parathyroid glands, bone and kidneys [93]. In the parathyroid glands, CSR expression is upregulated by vitamin D [120], and CSR activation inhibits synthesis and secretion of PTH [93]. In bone, CSR inhibits osteoclast activity and stimulates osteoblast activity, causing a diminished release of Ca^2+^ [121]. In the kidney, CSR is expressed in the luminal aspect of the proximal tubule and collecting duct and the basolateral aspect of the thick ascending limb and distal convoluted tubule (Figure 1) [94]. CSR activation inhibits renal Ca^2+^ reabsorption, acidifies urine and simultaneously causes salt and water wasting. Gain-of-function [122,123] and loss-of-function [124,125] mutations in CSR cause a spectrum of defects in Ca^2+^ homeostasis, parathyroid function and fluid regulation. 

Aging and CKD can have profound effects on both extrarenal and renal CSR by altering Ca^2+^ homeostasis. In the parathyroid glands, hypocalcemia seen in elderly CKD patients diminishes CSR signaling and releases CSR-mediated PTH inhibition, thereby enhancing PTH synthesis and secretion and promoting parathyroid hyperplasia. In bone, diminished CSR signaling can cause an accelerated bone resorption and attenuated bone formation and chondrogenesis [126]. In the kidney, a diminished CSR signal favors tubular Ca^2+^ × P_i_ precipitation and fluid retention. 

## 8. Mineral and Bone Disorder

MBD in elderly CKD patients is common and is etiologically related to altered balance in blood Ca^2+^ and P_i_. It is a systemic disorder characterized by one or more of the following [127]:

1. Laboratory abnormalities (*i.e.*, serum Ca^2+^ and P_i_, PTH and/or vitamin D).
2. Abnormalities in bone turnover, mineralization or volume.
3. Vascular or other soft tissue calcification.

Bone abnormalities in CKD-MBD range from the more common high-turnover osteitis fibrosa to low-turnover adynamic bone disease [128]. Mixed bone disease displays features of both high- and low-turnover. PTH, through its major downstream regulators osteoprotegerin (OPG) and receptor activator of NFκB-ligand (RANK-L), plays a major role in accelerating bone turnover. When RANK-L engages RANK, it activates osteoclasts, causing bone resorption. Osteoprotegerin (OPG) is a decoy for RANK-L and when engaged inactivates osteoclasts, decreasing bone resorption [129]. PTH simultaneously stimulates RANK-L expression and inhibits OPG production [130,131]. Patients with diabetes [132] and those on peritoneal dialysis [133] are predisposed to adynamic bone disease, associated with reduced PTH (<150 pg/mL), bone cellular activity and turnover [134]. These patients are at a high risk for hypercalcemia, due to a lack of bone buffering of the circulating Ca^2+^. Both the high- and low-turnover varieties of CKD-MBD are associated with increased risk of fracture, bone symptoms (e.g., bone pain), Ca^2+^ × P_i_ elevation, soft tissue and vasculature calcification and overall mortality [135]. Osteomalacia, featuring large amounts of osteoid material with deficient mineralization, occurs much less frequently in elderly CKD patients [136].

Duel-energy X-ray absorptiometry (DEXA) is routinely used clinically for assessing bone mineral density; its utility in CKD is limited as it fails to provide information on bone quality and architecture [137]. Biopsy of trabecular bone for histomorphometric analysis is the gold standard for diagnosis and ongoing evaluation of CKD-MBD [138]. In patients with high-turnover bone disease, there is trabecular thickening, increased resorptive activity, active mineralization and a background of marrow fibrosis. Marrow fibrosis can contribute to anemia and erythropoietin resistance. Excessive resorptive activity can cause release of minerals from the bone, promoting extraskeletal Ca^2+^ × P_i_ precipitation. In severe high-turnover MBD, normal lamella structure turns to a woven structure with haphazard organization [139]. At the other extreme, adynamic bone disease, bone tissue can show normal or thickened trabeculae with diminished or absent cellular activity. Tetracycline-labeling can be used to assess the pace of bone turnover [140].

Extraskeletal calcification, including vascular calcification, is an integral part of CKD-MBD. Both aging and CKD are intimately associated with the development and progression of soft tissue calcification. Incidence and progression of vascular calcification in the elderly is inversely related to bone mass [141,142]. Moreover, bone mineral loss and vascular calcification are prominent risk factors of fracture and all-cause mortality [143]. CKD in the elderly is a profound risk factor for the development of vascular calcification and vascular aging. Vascular calcification is evident in up to 94% of pre-dialysis CKD patients [144,145] and consistent with CKD patients dying from early onset of age-associated diseases, e.g., cardiovascular disease [146] and diabetes mellitus [147]. CKD is recognized as a model of premature aging [148]. 

Multiple mechanisms have been implicated in the genesis of vascular calcification and aging in CKD. In addition to hyperphosphatemia-mediated osteogenic transformation of soft tissue, uremic milieu can provoke phenotypic transition of vascular smooth muscle cells from the contractile phenotype to osteoblast-like phenotype [49]. This process is promoted by multiple factors, including osteoblastic morphogens (*i.e.*, the bone morphogenetic protein-2 and -4), core-binding factor α1 (also termed RUNX-2) and bone-related proteins, such as alkaline phosphatase and osteocalcin [149]. A recent study also shows that Klotho deficiency can trigger vascular calcification [150]. 

Vascular calcification, a strong risk factor for mortality, is likely a dynamic process and can potentially be modified. Sigrist *et al*. investigated the progression of vascular calcification over a two-year period in a cohort of CKD (60 hemodialysis, 28 peritoneal dialysis and 46 stage 4 CKD) patients [151]. There was an increase in the radiological evidence of vascular calcification in the two-year interval. The increase was associated with a widened pulse pressure and elevated pulse-wave velocity. The mortality during the study period was 42% in hemodialysis, 33% in peritoneal dialysis and 14% in stage 4 non-dialysis CKD. Increase in calcification score and Ca^2+^ intake (from P_i_ binders) and reduction in baseline plasma albumin were associated with reduced survival. Intriguingly, one-third of the patients in the three subgroups remained free of vascular calcification, and a small percentage (4%–8%) of the patients demonstrated regression. These findings suggest a dynamic process of vascular calcification that could potentially be modified, providing rationale for early intervention and aggressive control of calcification risks in CKD. 

## 9. Recommendations

Current guidelines from KDOQI (Kidney Disease Outcomes Quality Initiative) are that vitamin D deficiency in patients with CKD should be treated as in the general population [136]. Ca^2+^ intake in elderly CKD patients should generally be approximately 800–1000 mg daily. Overzealous Ca^2+^supplementation should be avoided. For CKD patients with evidence of secondary hyperparathyroidism, regular monitoring of serum Ca^2+^, P_i_, PTH and vitamin D is necessary. For patients with stages 3–4 CKD, serum Ca^2+^ should be maintained within normal range, 8.9–10.1 mg/dL, P_i_ should be within 2.7–4.6 mg/dL and PTH within 100–200 pg/mL. For patients with stage 5 CKD, Ca^2+^ should also be kept at normal range, P_i_ target should be <5.5 mg/dL and PTH in the range of 150–300 pg/mL. These targets can potentially be achieved with dietary modification and the appropriate use of P_i_ binders, vitamin D (*i.e.*, active vitamin D or analogues) and calcimimetics (*i.e.*, cinacalcet) [152], alone or in combination. 

Although P_i_ binders are routinely used in dialysis patients, for non-dialysis CKD patients, when to initiate them is uncertain. Moreover, whether there is any advantage to using Ca^2+^-free over Ca^2+^-containing preparations is unclear. A randomized, double-blind trial by Block *et al.* compared calcification scores for late CKD patients (eGFR: 20–45 mL/min/1.73 m^2^) on P_i_ binders (aggregate of Ca^2+^-containing and Ca^2+^-free binders) *versus* placebo for six months [153]. Despite P_i_ reduction, P_i_ binders failed to reduce vascular calcification scores. The study was not powered to differentiate calcification scores between Ca^2+^-containing and Ca^2+^-free P_i_ binders. Chue *et al*. investigated a cohort of non-diabetic early CKD patients (mean eGFR: 50 mL/min/1.73 m^2^) randomly assigned to receive Ca^2+^-free P_i_ binder (sevelamer) (*n* = 55) or placebo (*n* = 54) for 40 weeks [154]. Only 56% of the sevelamer group achieved >80% compliance, and these patients were analyzed. They found a reduction in serum FGF23 and urinary P_i_ excretion, however there was no reduction in cardiovascular-related outcomes. Further investigations with a better drug preparation to enhance compliance, larger patient cohort and longer study duration can be informative.

Vitamin D analogues (doxercalciferol and paricalcitol) are commonly used for controlling secondary hyperparathyroidism; paricalcitol has a lesser hypercalcemic and hyperphosphatemic effect [72,155]. Calcimimetic CSR blocker (cinacalcet) has also been introduced in recent years (FDA-approved in March, 2004) for treating severe (PTH > 600 pg/mL) secondary hyperparathyroidism [156]. The EVOLVE (evaluation of cinacalcet HCL therapy to lower cardiovascular events) trial demonstrated efficacy of cinacalcet in PTH reduction in dialysis patients with pre-therapeutic median PTH of ~690 pg/mL. After adjusting for baseline characteristics, cinacalcet was associated with reduced cardiovascular mortality [157]. Without the data adjustments, however, cinacalcet failed to show a survival advantage. The study also revealed intolerance to cinacalcet (primarily hypocalcemia, nausea and vomiting) in nearly a third of the patients. Thus, apart from PTH reduction, the clinical value of cinacalcet in CKD remains uncertain. 

## 10. Concluding Remarks

Elderly CKD population is on the rise. Ca^2+^ homeostasis is altered in the majority of these patients, manifested predominantly as hypocalcemia, hyperphosphatemia, vitamin D deficiency, FGF23 elevation (coupled with Klotho deficiency) and secondary hyperparathyroidism. These defects are deleterious to bone and soft-tissue health, leading to the development of MBD, which is associated with morbid clinical outcomes, including fracture, cardiovascular events and all-cause mortality. Recent studies suggest that vascular calcification in CKD might be prevented and/or ameliorated by correcting the Ca^2+^ alterations and vitamin D deficiency and by optimizing Ca^2+^ × P_i_ product levels. A guideline (KDOQI)-driven multi-disciplinary approach involving nephrologists, primary care physicians, physician assistants/extenders, nurses, dietitians and pharmacists for managing elderly CKD patients with Ca^2+^ dysregulation and MBD can be efficient, effective and is advisable.
